# Blame through my eyes: How first-person perspective shapes social attention and attribution in harmful interactions – a comparative eye tracking study

**DOI:** 10.1186/s40479-026-00348-0

**Published:** 2026-05-18

**Authors:** Adrianna Jakubowska, Raphaëlle Fortin, Makar Varenyk, Li Ting Lin, Anna Zajenkowska

**Affiliations:** 1 Research Institute of Psychology, VIZJA University, Szczęśliwicka 40, Warsaw, 02-353 Poland; 2 Department of Psychology, University of Montréal, Montreal, Canada; 3 Institute of Brain Science, National Yang Ming Chiao Tung University (NYCU), Taipei, Taiwan

**Keywords:** Social cognition, Perspective-taking, Eye-tracking, Violent offenders

## Abstract

**Background:**

Rigid attributional patterns are a key component of social cognitive deficits linked to aggression. Prior eye-tracking research using a third-person perspective revealed that, compared to community adults, incarcerated violent offenders oriented less to faces and showed a stronger correlation between perceived intentionality and assigned blame (Intentionality/Blame isomorphism), a metric of attributional inflexibility. The present study investigated how adopting an immersive first-person victim perspective affects both I/B isomorphism and facial attention.

**Methods:**

A total of 184 adults participated in our study, including 119 community members (60 female, 59 male) and 65 incarcerated violent offenders (32 female, 33 male). During the eye-tracking task, participants viewed 27 static visual scenes depicting ambiguous interpersonal harm between two actors - a harm doer and a victim - and were instructed to imagine themselves as the victim in each scenario. The scenes included both hostile (e.g., a harmful act) and non-hostile (e.g., mitigating expressions or gestures) cues. Independent samples t-tests were used to compare group differences in I/B isomorphism and face dwell time, and Pearson’s correlations were used to examine the relationships between variables.

**Results:**

Consistent with our hypothesis and contrary to findings from third-person studies, incarcerated violent offenders exhibited significantly lower I/B isomorphism than community adults under a first-person perspective. Furthermore, no significant differences in face dwell time were observed between these two groups.

**Conclusions:**

These results suggest that, among incarcerated violent offenders, adopting a victim’s perspective may disrupt rigid attributional patterns and normalize attention to social cues. These findings suggest that perspective-taking interventions could support improvements in empathy, cognitive flexibility, and overall interpersonal functioning.

## Background

### Social cognition as a foundation for interpersonal relationships

Social cognition refers to the set of cognitive abilities that enable individuals to infer intentions, feelings, and thoughts, as well as to interpret and judge their own and others’ social behaviors [2, 7]. It encompasses the fundamental mental operations that link internal processing to observable social behavior [28, 47]. This view is consistent with broader social cognitive frameworks, which emphasize mentalizing capacities and the role of medial frontal regions in representing others’ mental states and guiding social behavior [3, 23]. One prominent theory that explains these processes is the Social Information Processing (SIP) model [11, 16]. This theory posits that social behavior is the result of a six-step sequence of cognitive processes that individuals engage in when faced with a social stimulus. The first two steps - encoding social cues and their interpretation - lead to the formation of a mental representation of the social situation [11, 16]. Consequently, it is argued that deficits or biases at these initial stages play a crucial role in the development of maladaptive interpersonal behaviors, such as aggression [11]. From a social cognitive neuroscience perspective, such multi-stage processing reflects how social perception and mental state inference are implemented in distributed neural systems supporting social cognition [39]. According to the SIP model, individuals selectively attend to specific social stimuli. In social interactions, one of the most salient social cues is the human face [35, 62]. Facial features and expressions are among the primary pieces of information individuals selectively attend to when interpreting a social situation [38]. To measure these visual attention patterns, researchers use eye-tracking technology, which records eye movements to determine where a person is looking [17, 26]. Individuals with lower levels of personality functioning and those exhibiting psychopathic traits have shown reduced attention to faces and the eye region, quantified by shorter dwell times [26, 64]. Such processing of facial cues may impact the interpretation of these cues [11]. Individuals with psychopathic traits or violent offenders have well-documented deficits in facial affect recognition, particularly regarding the identification of fear, and in some studies also happiness [8, 10, 42]. Recent neuroimaging work further indicates heightened amygdala reactivity to angry faces in individuals with antisocial personality disorder, consistent with an increased sensitivity to anger-related social threat [36]. These emotion-processing abnormalities are often accompanied by an increased sensitivity to angry facial expressions and a tendency to over-perceive anger in ambiguous or neutral faces ([44]; https://doi.org/10.1016/j.paid.2008.01.004 [31]). This altered emotional salience suggests that threat-related stimuli, such as anger, may more readily capture attention and shape interpretation processes in offender populations, consistent with models of hostile attribution bias [37, 53]. Eye-tracking and neuroimaging findings further suggest that antisocial populations may preferentially orient toward angry cues while showing reduced sensitivity to other emotional signals, a pattern that may reinforce hypervigilance and reactive aggression [36]. This tendency to interpret ambiguity as hostile is part of a broader pattern of cognitive distortions known as hostile biases. These biases include also Hostile Attribution Bias (HAB) and Hostile Perception Bias (HPB; [53]). HAB can be further subdivided into Relational HAB (HAB-R), triggered by relational provocations such as social exclusion or being ignored, and Instrumental HAB (HAB-I), elicited by instrumental provocations like being physically pushed or bumped [12, 13, 27].

### Intentionality/blame isomorphism

The process of HAB typically involves three factors: the attribution of intentionality, anger experience, and the ascription of blame. Although intentionality and blame are theoretically distinct constructs [68], some individuals struggle to differentiate between them, which may reflect some attributional inflexibility or rigidity [65]. It is important to clarify that ‘cognitive rigidity’ is a broad construct [24]. Our study focuses on the ‘process rigidity’ that occurs after an initial appraisal of intent is made. We operationalize this ‘process rigidity’ as Intentionality/Blame isomorphism (I/B isomorphism): the statistical correlation between these two factors [65]. A higher I/B isomorphism suggests a more automatic and inflexible assignment of blame once intentionality is perceived, implying a reduced capacity to process broader contextual cues and mitigating information. Specifically, a previous study found higher I/B isomorphism in incarcerated violent offenders, as compared to community individuals, when assessing harmful scenarios involving others (i.e., using a third-person perspective; [65]). Higher I/B isomorphism in scenarios involving others - when participants assessed a harmful event in which they were not directly involved - is associated with reduced attention to faces, suggesting that impairments in interpreting social situations are linked to specific patterns of social cue encoding [65]. 

Furthermore, the perception of intentionality and blame as overlapping constructs may prevent individuals from recognizing mitigating circumstances, considering others’ arguments, or accepting differing viewpoints, thereby perpetuating cycles of conflict. For example, an individual might perceive another person’s action as intentional harm; however, multiple alternative explanations could exist for that behavior [41, 45, 65]. The attribution of blame would therefore depend on how the observer interprets the actor’s intentions and the contextual factors surrounding the event [65]. 

A critical factor in attributional processes is whether a situation is perceived as a personal threat [14], particularly in evaluating behaviors directed toward oneself. Due to the unique nature of the prison environment, incarcerated offenders must be particularly careful in their social judgments [30, 63]. They live in a setting where misinterpreting intentions, and particularly misattributing blame to behaviors directed toward themselves, can have immediate and severe consequences [29]. Prisoners, therefore, may think longer before assigning blame, weighing the potential consequences of perceiving an act as fully blameworthy. While prior findings indicate higher I/B isomorphism in third-person scenarios [65], adopting a first-person victim perspective - even in the high-stakes prison environment - may potentially disrupt this automatic rigid pattern by prompting a shift from automatic to reflective processing [31]. This aligns with social cognition literature distinguishing third-person perspectives, which foster detached, automatic judgments [18], from more immersive first-person perspectives, which promote self-referential engagement and can support less egocentric, jointly coordinated forms of understanding [57]. The individual’s position (i.e., whether they are directly involved in the harmful situation or merely observing it) may thus prompt greater differentiation between intent and blame, manifesting as lower I/B isomorphism [32].

### Violent behavior and interpersonal difficulties

Understanding the mechanisms of social cognitive rigidity, such as I/B isomorphism, is important as these processes are theorized to be foundational to real-world interpersonal outcomes. Deficits in cognitive flexibility can undermine the development and maintenance of interpersonal relationships and broader interpersonal functioning [1, 21, 34]. Indeed, one of the main corelates of criminal and violent behavior is poor interpersonal functioning [33, 46]. Therefore, investigating a core mechanism like I/B isomorphism may provide insight into the specific cognitive dysfunctions that are hypothesized to contribute to the profound interpersonal difficulties observed in violent offender populations [59].

### Current study

Prior research investigating attributional rigidity found that offenders exhibit high I/B isomorphism when using a third-person perspective [65]. A third-person vantage point tends to foster a detached, judgmental, and evaluator-like stance, in which the observer remains outside the situation and relies on more automatic, rigid attributional processes [18]. While a detached perspective may facilitate an offender’s automatic and rigid cognitive style, the instruction to imagine oneself as the victim is expected to encourage deeper, self-referential processing and promote less egocentric judgments [32]. More broadly, adopting a first-person victim perspective aligns with accounts of shared intentionality, in which taking another’s viewpoint involves coordinating one’s own and others’ mental states within a common psychological frame [57, 58]. Thus, at a theoretical level, third-person perspective emphasizes evaluative distance, whereas an immersive first-person victim perspective emphasizes self-referential engagement with the harmful event and the other’s mind. Adopting an immersive first-person victim perspective may increase attention to social cues as individuals attempt to understand the harm being directed at them, potentially disrupting the automatic link between perceived intent and assigned blame [65]. Furthermore, the first-person victim instruction may function as an empathic induction [43, 64]. Also, empathy itself can act as a cognitive inhibitor of automatic, aggressive interpersonal responses [49]. Moreover, in the threatening prison environment, making a rapid and automatic blame judgment is socially costly and potentially dangerous [41, 45, 54]. By forcing a self-relevant (victim) perspective, the task likely engages this more cautious processing, slowing down the judgment. This forces a differentiation between the cognitive steps: the initial appraisal ‘Did they mean it?’ (Intent) is separated from the more costly, high-stakes question, ‘Are they to blame?’ (Blame). We therefore reasoned that this induced reflective state would disrupt the rigid cognitive link, forcing a more considered differentiation between intent and blame. This greater differentiation would, in turn, manifest as a statistically lower I/B isomorphism score. This capacity for perspective-taking to improve social cognition may be supported by a study on violent offenders in this area that used virtual reality to place domestic abusers in a female victim’s perspective. This first-person view significantly improved subsequent fear recognition in the violent offender group, but not in the control group [52]. However, this study had a small, all-male sample consisting of 20 offenders and 19 controls. Extending this line of research, we investigated whether adopting a self-referent perspective (i.e., imagining oneself as the harmed person in a social event) would lead violent offenders, compared to community participants, to exhibit reduced I/B isomorphism. Accordingly, we hypothesized that violent offenders would demonstrate lower I/B isomorphism than community participants (H1). Furthermore, replicating previous findings [65], we hypothesized that dwell time on faces would be negatively associated with I/B isomorphism (H2).

In addition, we conducted an exploratory analysis of the relationships among I/B isomorphism, dwell time on faces, blame, and intentionality in both groups: male and female violent offenders, and individuals with no history of violence.

## Methodology

### Participants and procedure

The study sample consisted of 184 individuals from two groups: a community sample and offenders convicted for violent crimes. The community sample included 60 women (mean age = 37.44 years, SD = 11.4) and 59 men (mean age = 37.5 years, SD = 11.2). Incarcerated violent offender group consisted of 32 women (mean age = 36.4 years, SD = 8.74) and 33 men (mean age = 38.94 years, SD = 8.84). The reported duration of incarceration was M = 7.07 years (SD = 6.88), with a range of 2.5 months to 25.0 years. 16.8% of the community sample had attended or completed higher education, 35.3% had a secondary education, and 46.2% had an education below the secondary level. In the offender group, 1.5% of participants had attended higher education, 24.6% had a secondary education, and 72.3% had an education below the secondary level.

Our study was powered to detect a conventional medium effect size (*d* = 0.50). An a priori power analysis using G*Power [20] indicated that a total sample of *N* = 128 (64 per group) would be needed for 80% power. Our final recruited sample (*N* = 184; N1 = 119, N2 = 65) provided 89.7% power to detect a medium effect size (*d* = 0.50) at an alpha of 0.05 (two-tailed). Therefore, the study was adequately powered. 

The study was a part of a larger project. Recruitment for the two groups was conducted separately. The community sample (*N* = 119) was recruited from the general population using online advertisements. For this group, we inquired about participants’ past imprisonments, and only those without any previous imprisonment were included in the study. The incarcerated violent offender sample (*N* = 65) was recruited from one prison with the assistance of the local prison psychologist. Eligibility for the offender group was restricted by prison authorities to individuals incarcerated for violent offenses against persons (e.g., assault, homicide, robbery; Quinsey et al. [47]. In both groups, an additional exclusion criterion was insufficient or uncorrected eyesight that could interfere with eye-tracking calibration or data quality. Prior to data collection, participants were thoroughly briefed on the study’s objectives, its voluntary nature, and the guarantee of complete anonymity. Sessions were conducted individually in quiet, private rooms (either at the eyetracking lab for the community sample, or at the prison facility for the offender sample) and were administered by a trained psychologist. Following this briefing, each individual first completed a battery of self-report questionnaires (including demographic questions used for sample characterization; clinical measures such as the PHQ-9 for depression were collected but not analyzed for this study), and then completed the eye-tracking visual scenes task. Upon completion of the study, every participant was awarded a 30 PLN voucher.

### Ethics approval and consent to participate

The research received ethical approval from the Maria Grzegorzewska University ethics committee (approval #123/2022). All procedures performed in this study were in accordance with the ethical standards of the institutional research committee and with the 1964 Helsinki Declaration and its later amendments. Written informed consent to participate was obtained from all individual participants included in the study.

### Funding

This research was funded by the National Science Centre, Poland, grant number UMO- 2017/26/D/HS6/00258 and partially by 2021/42/E/HS6/00018.

### Measures

**Visual scenes** [60, 66–68] - consisted of 27 previously validated pictures depicting scenes with two actors - a harm doer (perpetrator) and a harm receiver (victim). Participants were asked to take a victim’s perspective (first-person perspective) in each scenario. Scenes depicted peers interacting with each other, or subordinates and authority figures. All 27 scenes were intentionally ambiguous, designed to contain both hostile (the harmful act) and non-hostile (e.g., a smile, mitigating body language) cues. The main task was preceded by 2 practice trials to ensure participants understood the instructions. Each scene was displayed on a computer screen for 6 seconds. On a subsequent screen, participants first made intentionality [“Please rate to what extent this act was intentional” on a Likert scale ranging from 1 (not intended at all) to 9 (intended)] and then blame judgments [“Please rate to what extent you would blame the person for that” (1 – not at all; 9 – very much); see [66]]. No scheduled breaks were included. Intentionality/blame isomorphism was calculated in R as a within-person Pearson correlation coefficient between intentionality and blame ratings across all scenes.

### Eye-tracking

Eye movements were measured using a Tobii Pro X3-120 remote eye tracker with a sampling rate of 120Hz, and the binocular accuracy 0.50/precision 0.250. Data recording was done using a Dell laptop. Stimuli were presented on a 17^’’^ computer screen (1920 × 1080 pixels resolution) for 6 s. Participants viewed the presented scenes from a distance of ca. 60–70 cm from the screen. The study was preceded by a 9‐point calibration, repeated until a good calibration result was obtained.

### Availability of data and materials

The dataset supporting the conclusions of this article is available in the Open Science Framework repository, https://osf.io/9hxwa/?view_only=5f79d21f15ba4a77a88e47763853df51.

## Data analysis

### Behavioral data

In addition to I/B Isomorphism, we computed two other person-level indices from the behavioral ratings to deconstruct our primary finding (H1) and better understand the qualitative nature of the observed group difference in attributional rigidity. We created a ‘Response Consistency’ index for each participant, calculated as the standard deviation of their Blame-Intent difference scores across all trials; a higher score indicates greater inconsistency. We also computed a ‘Mean Blame-Intent Difference’ score for each participant (Mean(Blame - Intentionality)) to assess the average tendency to mitigate blame relative to intent.

### Eyetracking data

To measure attention to faces, faces of both the harm doers and harm receivers were defined as a single, combined area of interest (AOI; a frame including whole faces) using iMotions software (version 8.1). We used this aggregated AOI to test our general hypothesis and maintain consistency with our prior work [65]. Eye-tracking data were pre-processed within iMotions. Fixations were identified from the raw gaze data using the Identification by Velocity Threshold (IVT) algorithm [50]. Dwell time (FDT) was then calculated as the total duration of all qualified fixations within the AOI, excluding data points between fixations.

### Data cleaning and exclusions

Trials with missing behavioral ratings (i.e., a participant failed to answer) were excluded. The I/B isomorphism coefficient could only be computed for participants who provided variance in both their intentionality and blame ratings. In total, five participants were excluded from all analyses involving the I/B isomorphism variable due to insufficient data.

## Results

Descriptive statistics (means and standard deviations) for all study variables by group are reported in Table 1.

**Table 1:** Means and standard deviations for all study variables by group

Variable	Community Adults (*N* = 119)	Violent Offenders (*N* = 65)	Group Comparison
I/B Isomorphism (ISO)	0.60 (0.32)	0.46 (0.45)	*p* = .008, *d* = 0.38
Intentionality (INT)	6.08 (1.13)	5.89 (1.33)	*p* = .304
Blame (BLM)	5.58 (1.33)	4.81 (1.91)	*t*(181) = 3.21, *p* < .001, *d* = 0.50
Face Dwell Time (FDT)	1378.59 (445.48)	1356.91 (463.91)	*p* = .758

### Group differences in I/B isomorphism and face dwell time (H1)

**Fig. 1:**
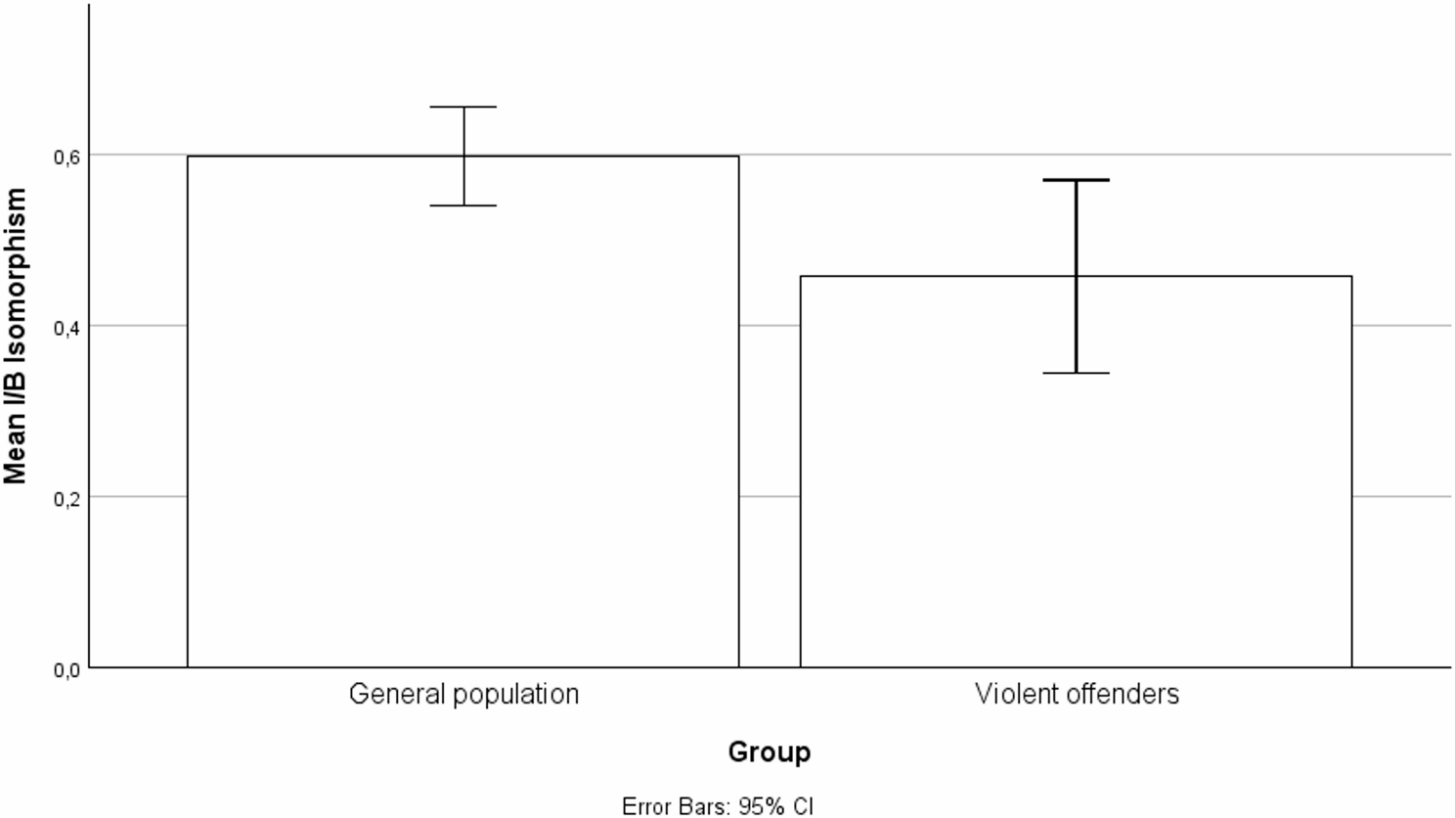
Mean intentionality/blame isomorphism (I/B isomorphism) by group, displaying 95% confidence intervals

To investigate differences between the violent offender group and the community adult group in intentionality/blame isomorphism (H1) and, additionally, in dwell time on faces, independent-samples t-tests were conducted. Consistent with H1, violent offenders exhibited lower I/B isomorphism than the community group (*p* = .008, *d* = 0.38; Fig. 1). However, no significant group differences were observed in dwell time on faces (*p* = .758).

### Control analyses for group difference in isomorphism

Given the significant differences in educational attainment between the groups, we conducted a follow-up hierarchical linear regression analysis to ensure this did not confound the finding for H1. With I/B Isomorphism as the dependent variable, we entered Education Level as a predictor in Step 1. This model was not significant (*F*(1, 177) = 0.006, *p* = .939). In Step 2, we added Group (Offender vs. Community) to the model. The addition of Group explained a significant 3.6% of new variance (*ΔR²* = 0.036, *F-change*(1, 176) = 6.52, *p* = .012). These results indicate that the group difference in I/B isomorphism remains significant.

To explore the potential influence of gender on our findings, we conducted an exploratory 2 (Group: Offender, Community) x 2 (Gender: Female, Male) factorial ANOVA on I/B Isomorphism. The analysis revealed a significant main effect of Group (*F*(1, 177) = 5.97, *p* = .016), confirming our primary finding. There was no significant main effect of Gender (*F*(1, 177) = 0.39, *p* = .532). Crucially, the Group x Gender interaction effect was not significant (*F*(1, 177) = 1.91, *p* = .169). This indicates that the observed group difference in I/B Isomorphism was consistent across both male and female participants.

### Underlying mechanisms of isomorphism

Before comparing group means on I/B Isomorphism, we conducted Levene’s Test for Equality of Variances. The test was significant (*F* = 7.96, *p* = .005), indicating that the variance of I/B isomorphism scores in the offender group (SD = 0.45) was significantly greater than in the community group (SD = 0.32). This suggests that the offender group is substantially more heterogeneous in their attributional styles.

To investigate the nature of the observed isomorphism difference, we compared the groups on their ‘Response Consistency’ index. A Welch two-sample t-test revealed that the offender group (*M* = 2.54) was significantly more inconsistent in their response patterns from scene to scene than the community group (*M* = 1.94), *t*(96.9) = -2.65, *p* = .009.

Furthermore, we examined the Mean Blame-Intent Difference. An independent-samples t-test was conducted to directly compare the magnitude of this discrepancy between the groups. Levene’s test indicated unequal variances (*F* = 18.96, *p* < .001). The analysis confirmed that the tendency to rate blame lower than intent was significantly more pronounced in the violent offender group than in the community group, *t*(84.92) = 2.61, *p* = .011, *d* = 0.47. This finding may appear counterintuitive but aligns with the lower I/B isomorphism in offenders. Offenders showed greater response heterogeneity (SD = 0.45 vs. 0.32), with divergent styles: some exhibited rigid blame-intent coupling (driving ISO-BLM *r* = .418***), while others showed extreme decoupling (larger gaps). Community adults were more homogeneous, with consistently strong INT-BLM coupling (*r* = .696***).

### Correlational analyses (H2)

**Fig. 2:**
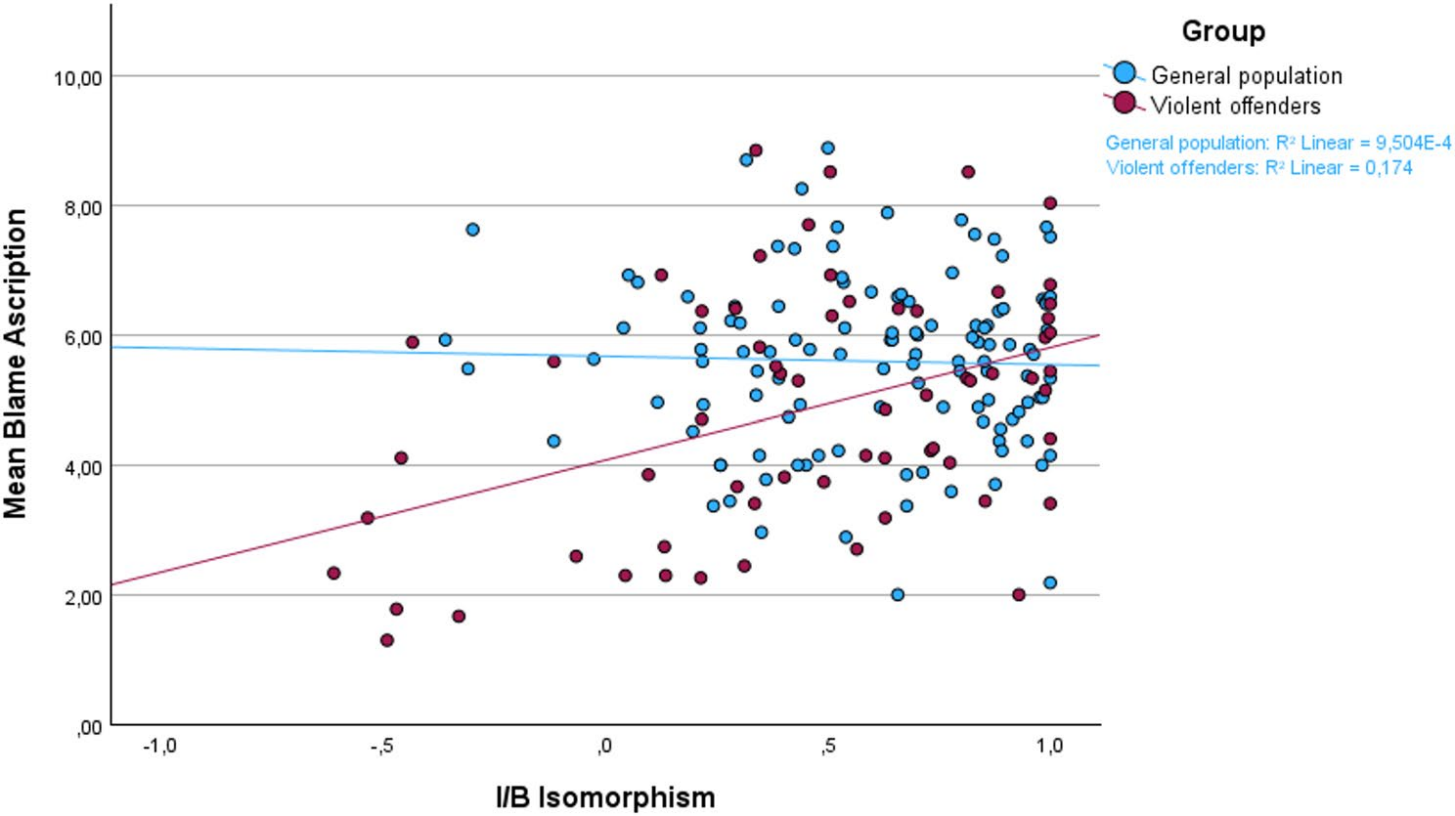
Relationship (regression lines) between I/B isomorphism (x-axis) and mean blame (y-axis) for the general population and violent offender groups

Correlational analyses were conducted to examine the relationships between I/B isomorphism and dwell time on faces (H2), and also other studied variables, including blame and intentionality ascription (see Table 2; Fig. 2).

**Table 2:** Pearson’s r coefficients for numeric study variables by group and for the whole sample

	Whole sample	Community adults	Violent offenders
ISO – INT	−0.072	− 0.181*	0.01
ISO – BLM	0.231**	−0.031	0.418***
ISO – FDT	− 0.227**	− 0.265**	−0.198
INT – BLM	0.579***	0.696***	0.455***
INT – FDT	−0.052	−0.008	-0.124
BLM – FDT	−0.063	−0.05	-0.092

Note. * *p* < .05, ** *p* < .01, *** *p* < .001; ISO – Isomorphism, INT – Intentionality, BLM – Blame, FDT – dwell time of faces

Across the entire sample, higher I/B isomorphism (ISO) was associated with lower attention to faces (FDT), which is consistent with H2. However, subgroup analyses revealed distinct patterns for each population. Among community adults, higher I/B isomorphism was significantly correlated with both lower attention to faces (FDT) and lower attributions of intentionality (INT). In contrast, the link between I/B isomorphism and face attention was non-significant for violent offenders. Instead, in this group, higher I/B isomorphism showed a strong, significant positive correlation with blame (BLM).

### Additional correlational analyses

Finally, we correlated our new person-level indices. We found a very strong negative correlation between I/B Isomorphism and Response Consistency (*r* = -.82, *p* < .001), suggesting that lower isomorphism is largely a function of higher response discrepancy. Also, we found a significant positive correlation between I/B Isomorphism and the Mean Blame-Intent Difference (*r* = .37, *p* < .001).

## Discussion

The present study explored differences in intentionality/blame isomorphism between violent offenders and community adults when adopting a first-person perspective – that is, imagining oneself as the harmed person in a social event. We also examined attention to faces and tested whether, under such conditions, I/B isomorphism would remain negatively correlated with dwell time on faces.

Previous research has indicated that offenders have been shown to exhibit a stronger overlap between perceived intent and blame (I/B isomorphism) when interpreting ambiguous social scenarios from a third-person perspective [65]. In contrast, the current study found that when instructed to adopt the victim’s point of view (a first-person perspective), violent offenders exhibited significantly lower I/B isomorphism than community adults. While this effect was of a small-to-moderate size, it suggests that the context of perspective-taking may be a meaningful factor in this sample. This finding is consistent with the victim–perpetrator asymmetry, which proposes that victims interpret harmful actions as more intentional and damaging, whereas perpetrators tend to downplay their impact [6]. Adopting a victim perspective in a task requires offenders to shift from a perpetrator to a victim lens, confronting them with a viewpoint that highlights harm and intentionality. This shift, in turn, may encourage greater caution in attributing intentionality and blame. These findings suggest that the typically rigid link between perceived intent and assigned blame may be disrupted through brief perspective-taking instructions, at least under controlled experimental conditions. Consequently, violent offenders’ attributions may be highly context-dependent, which has potential implications for interpersonal functioning. By adopting a first-person perspective, aggressive individuals may momentarily disrupt their automatic, rigid processing, becoming less likely to respond with immediate hostility [1, 21, 34].

Our additional analyses suggest that I/B isomorphism may not be a unitary construct but rather reflects qualitatively different patterns across the two groups. Among community participants, higher rigidity was associated with lower attention to faces and lower intentionality ratings. This pattern suggests a consistent, perhaps more disengaged or norm-following processing style, where individuals rapidly categorize ambiguous situations without deeply evaluating social cues or attributing strong hostile intent. In contrast, violent offenders demonstrated a highly variable and sometimes strongly punitive style. Within this group, higher rigidity was strongly correlated with higher overall blame ascription and a smaller Mean Blame–Intent Difference. This indicates that for offenders, as cognitive rigidity increases, the capacity to consider mitigating circumstances decreases, forcing blame ratings to match perceived intent. Therefore, while community adults’ rigidity appears linked to a benign, disengaged heuristic, offenders’ rigidity seems driven by a punitive mechanism that tightly couples the perception of harm with the immediate assignment of blame. Consequently, the lower average I/B isomorphism observed in the offender group as a whole seems to reflect increased intra-individual response variability and noise across different social scenarios, rather than a stable, generalized improvement in cognitive flexibility. The exact mechanisms driving this increased variability and decoupling of intent and blame remain unknown. One speculative hypothesis for future research is that adopting the victim’s perspective may, for some incarcerated violent offenders, activate defensive processes that reduce the explicit acknowledgment of harm. When individuals perceive themselves as targets of wrongdoing, recognizing the full intentionality and blameworthiness of others may heighten psychological vulnerability and perceived interpersonal threat, a position some offenders may find difficult to tolerate. Dampening the link between intent and blame could then function as a regulatory strategy to protect against negative affect, anger escalation, or moral accountability [6, 40, 56]. These interpretations are speculative and should be treated as hypotheses for future research, for example studies that directly assess defensive processes, trauma history, and attachment patterns alongside I/B isomorphism.

Furthermore, when participants were asked to take the victim’s perspective, there were no significant differences in visual attention to facial cues (face dwell time) between violent offenders and community adults. Yet when using a third-person perspective, violent offenders showed significantly less attention to faces [65]. While these differences in attention may suggest that first-person instructions prompt incarcerated offenders to become more attuned, or at least as much as the community adults, to subtle social cues, either by noticing cues they might usually miss or by reevaluating those they might otherwise interpret as hostile – regulating attention toward facial expressions is considered one of the most effective strategies for supporting accurate social perspective-taking [25]. However, an alternative interpretation is that this null effect may be a function of the task design itself. Social attention deficits in offenders may be primarily triggered by dynamic, real-world social cues (e.g., changing facial expressions, body language). Our static images may not be engaging enough to elicit the baseline attention deficit in the first place. Our findings must therefore be interpreted with caution.

Our findings may challenge the notion that incarcerated violent offenders are fundamentally impaired in mentalization, which is the ability to understand and reflect on the thoughts, feelings, and intentions of oneself and others [22]. Rather, they suggest that offenders may simply be less likely to engage in this ability spontaneously due to a cognitive rigidity, i.e., the tendency to perseverate in the use of a mental or behavioral state [51], such as HAB and I/B isomorphism [65]. The instruction to adopt the victim’s perspective appears to be one way to activate controlled cognitive resources that enable the reinterpretation of ambiguous intentions [4, 60, 61]. We speculate that this ability may be particularly accessible for incarcerated violent individuals when they are prompted to engage it, given that many have experienced both sides of the violence dynamic: as victims and as perpetrators [15, 19]. This dual experience could paradoxically support more cognitive flexibility as they might draw on their lived experience to better mentalize each character (victim and persecutor) and, therefore, separate blame (assigned from the victim’s point of view) from intention (persecutor’s mental state). Indeed, mentalization can be facilitated by processes of self-projection, in which individuals draw on their own past experiences and use themselves as a point of reference to infer the mental states of others [9, 18, 55].

### Limitations

Although our results highlight the potential of perspective-taking to disrupt rigid attributional patterns in violent offenders, several limitations must be acknowledged. A primary limitation of the current study is the absence of a direct, methodological comparison between first- and third-person perspectives within the same experimental design. Because the main theoretical contribution of our work relies on contrasting our findings (first-person perspective) with those from prior studies utilizing a third-person perspective (e.g., [65]), conclusions regarding the specific impact of self-relevance must be drawn with caution. Without a within-subject or randomized between-subject design directly manipulating the adopted perspective, we cannot rule out that cohort or unmeasured sample differences contributed to the observed effects. Future research must employ such designs to confirm the causal role of perspective-taking in attributional rigidity.

It is also worth noting that although our research addresses cognitive mechanisms theorized to be foundational to interpersonal functioning, it does not directly measure interpersonal functioning itself. Our findings suggest that perspective-taking instructions can reduce cognitive rigidity and improve social perspective-taking [25], but we cannot determine whether these changes translate into improvements in offenders’ everyday relationships. A crucial next step is to confirm the relationship between cognitive rigidity and interpersonal functioning. Future studies should include specific measures, such as interpersonal life satisfaction or perceived relationship quality, to empirically test whether these observed, instruction-dependent changes in cognitive rigidity translate to improvements in interpersonal functioning.

Similarly, our study did not include measures of trait anger or the tendency toward aggressive responses, although committing a violent crime can be considered a behavioral indicator of such tendencies. Thus, we cannot conclude that violent offenders had a higher level of aggression or anger trait than the community adult group. Future research should verify whether the observed effects are moderated by the level of aggression or rumination tendencies.

It is worth noting that our study relied on static images, which do not fully capture the complexity of real-world social interactions. Crucial elements for social information processing-such as tone of voice, dynamics of facial expressions, or speaking rate-are absent in static pictures, meaning they may not be sufficient to elicit the attention deficits typically triggered by dynamic cues. Furthermore, our use of an aggregated area of interest (AOI)-which combined both actors’ faces to maintain consistency with prior work-may have further limited task sensitivity. This metric could mask more nuanced gaze patterns, such as differences in how attention is distributed between the perpetrator and the victim. Thus, our null findings for face dwell time should be interpreted with caution. Future research should prioritize more dynamic stimuli, such as video vignettes or immersive virtual reality (VR) paradigms, to measure attentional and attributional patterns with greater ecological validity.

## Conclusions

Our study’s results suggest that, among incarcerated violent offenders, adopting a victim’s perspective may disrupt rigid attributional patterns and normalize attention to social cues. This highlights the potential of targeted perspective-taking interventions to enhance cognitive flexibility and foster skills essential for improving interpersonal functioning in this group. From an applied standpoint, these interventions may most effectively take the form of psychoeducational programs grounded in Mentalization-Based Therapy (MBT) principles. As outlined by Bateman [5], MBT fosters empathy, interpersonal functioning, and awareness of how one’s actions affect others by strengthening the capacity for accurate self-reflection and emotional regulation. Consistent with this approach, Zajenkowska et al. [69] demonstrated that MBT-inspired psychoeducational exercises enhanced incarcerated participants’ ability to adopt others’ perspectives and search for alternative explanations when analyzing ambiguous social situations, suggesting increased flexibility in attributing intention and blame. Our findings are consistent with this framework, but future intervention studies are needed to establish whether perspective-taking instructions embedded in MBT-based programmes can causally reduce cognitive rigidity and improve interpersonal functioning in incarcerated violent offenders.

## Data Availability

The dataset supporting the conclusions of this article is available in the Open Science Framework repository, https://osf.io/9hxwa/?view_only=5f79d21f15ba4a77a88e47763853df51.
